# *Phospho1* deficiency transiently modifies bone architecture yet produces consistent modification in osteocyte differentiation and vascular porosity with ageing

**DOI:** 10.1016/j.bone.2015.07.035

**Published:** 2015-12

**Authors:** B. Javaheri, A. Carriero, K.A. Staines, Y.-M. Chang, D.A. Houston, K.J. Oldknow, J.L. Millan, Bassir N. Kazeruni, P. Salmon, S. Shefelbine, C. Farquharson, A.A. Pitsillides

**Affiliations:** a The Royal Veterinary College, London, United Kingdom; b Department of Biomedical Engineering, Florida Institute of Technology Melbourne, FL 32901, USA; c The Roslin Institute and R(D)SVS, University of Edinburgh, Edinburgh, United Kingdom; d Sanford Children's Health Research Center, Sanford‐Burnham Medical Research Institute, La Jolla, CA, USA; e Imperial College, London. United Kingdom; f Bruker-microCT, Kartuizersweg 3B, 2550 Kontich, Belgium; g Department of Mechanical and Industrial Engineering, Northeastern University, USA

**Keywords:** PHOSPHO1, Mineralisation, Osteoblast, Osteocyte, Vascular porosity, MicroCT

## Abstract

PHOSPHO1 is one of principal proteins involved in initiating bone matrix mineralisation. Recent studies have found that *Phospho1* KO mice (*Phospho1*-R74X) display multiple skeletal abnormalities with spontaneous fractures, bowed long bones, osteomalacia and scoliosis. These analyses have however been limited to young mice and it remains unclear whether the role of PHOSPHO1 is conserved in the mature murine skeleton where bone turnover is limited. In this study, we have used *ex-vivo* computerised tomography to examine the effect of *Phospho1* deletion on tibial bone architecture in mice at a range of ages (5, 7, 16 and 34 weeks of age) to establish whether its role is conserved during skeletal growth and maturation. Matrix mineralisation has also been reported to influence terminal osteoblast differentiation into osteocytes and we have also explored whether hypomineralised bones in *Phospho1* KO mice exhibit modified osteocyte lacunar and vascular porosity. Our data reveal that *Phospho1* deficiency generates age-related defects in trabecular architecture and compromised cortical microarchitecture with greater porosity accompanied by marked alterations in osteocyte shape, significant increases in osteocytic lacuna and vessel number. Our in vitro studies examining the behaviour of osteoblast derived from *Phospho1* KO and wild-type mice reveal reduced levels of matrix mineralisation and modified osteocytogenic programming in cells deficient in PHOSPHO1. Together our data suggest that deficiency in PHOSPHO1 exerts modifications in bone architecture that are transient and depend upon age, yet produces consistent modification in lacunar and vascular porosity. It is possible that the inhibitory role of PHOSPHO1 on osteocyte differentiation leads to these age-related changes in bone architecture. It is also intriguing to note that this apparent acceleration in osteocyte differentiation evident in the hypomineralised bones of *Phospho1* KO mice suggests an uncoupling of the interplay between osteocytogenesis and biomineralisation. Further studies are required to dissect the molecular processes underlying the regulatory influences exerted by PHOSPHO1 on the skeleton with ageing.

## Highlights

• Our data suggest that PHOSPHO1 deficiency exerts modifications in bone architecture that are transient and depend upon age.
• Phospho1 deficiency leads to increased osteocytic lacunar and vascular porosity.
• Phospho1 driven mineralisation is not coupled with osteocyte differentiation.

## 1 Introduction

Bone formation involves a cascade of events leading to the deposition of mineral (biomineralisation), critical to skeletal maintenance throughout life. Mineralisation occurs by a series a complex physico-chemical and biochemical processes that facilitate the deposition of a solid hydroxyapatite (HA) phase [1]. Biomineralisation can be considered a two-step process, which involves de novo induction of mineral formation within the protective enclave of the lumen of osteoblast and chondrocyte matrix vesicles (MVs) followed by propagation of induced mineral into the extravesicular matrix [2], [3]. These formation and propagation steps of HA deposition are carefully regulated by a balance of mineralisation promoters and inhibitors.

The recognised local inhibitors include inorganic pyrophosphate (PP*_i_*) and organic non-collagenous proteins or peptides of the extracellular matrix (ECM) such as osteopontin [4], [5], [6]. Bone mineralisation is also dependent on a tight local balance between extracellular (e) levels of P*i* and PP*_i_* and when ePP*_i_* is deficient or in excess, the skeleton is either over- or under-mineralised, respectively [7], [8]. The complex interplay between PP*_i_* formation, transport and degradation directly controls the eP*i*/PP*_i_* balance and thereby the propagation of HA out with the confines of the MV.

Current evidence suggests that there are several principal proteins involved in regulating bone mineralisation, which include tissue-nonspecific alkaline phosphatase (TNAP), an ectoenzyme expressed on the surface of chondrocytes, osteoblasts and their shed MVs [9]. TNAP hydrolysis maintains ePP*_i_* levels at physiological concentrations which also yields P*_i_* for HA formation within the ECM [10]. Nucleotide pyrophosphatase phosphodiesterase 1 (NPP1) also regulates mineralisation by generating PP*_i_* ectoplasmically from nucleoside triphosphate substrates [11], [12] whereas the multiple-pass transmembrane protein ANK achieves this by mediating intracellular to extracellular channelling of PP*_i_* [13], [14]. Mouse models with NPP1 or ANK mutations show decreased levels of PP*_i_* and bone hypermineralisation [14], [15]. PHOSPHO1 (phosphatase, orphan 1) which directly regulates PP*i* availability, has also now been identified.

TNAP deficiency in humans results in hypophosphatasia (HPP) and is linked to increased plasma PP*_i_* levels due to impaired pyrophosphatase function. Similarly, mice deficient in TNAP function (*Alpl^−/−^*) are born with normally calcified skeletons but by postnatal day 6 skeletal hypomineralisation becomes apparent and worsens with age until their early demise by postnatal day 20 [7], [16]. The failure of bones to calcify after birth appears to result from a block in HA propagation in the ECM, beyond the confines of the MV membrane [17], [18], as a consequence of accumulated ePP*_i_* levels due to lack of TNAP's pyrophosphatase activity [19], [20], [21] and concomitant pyrophosphate-induced increase in osteoblast production of osteopontin [22], [23]. Importantly, electron microscopy has revealed that MVs from *Alpl^−/−^* mice and from patients with hypophosphatasia possess the ability to initiate HA formation within the sheltered interior of the MV [24], [25]. These findings suggest that alternative mechanisms may regulate the intravesicular initiation of mineral formation. One candidate is PHOSPHO1, a soluble cytosolic phosphatase and a member of the haloacid dehalogenase (HAD) superfamily of hydrolases [26].

PHOSPHO1 was first identified in the chick where it is expressed at 120-fold higher levels in mineralising than non-mineralising tissues [27]. It is active in osteoblast and chondrocyte MVs and has specificity for phosphoethanolamine (PEA) and phosphocholine (PCho) [28], [29]. The reduced ability of the chick wing and leg long bones to mineralize in the presence of the PHOSPHO1 inhibitor, lansoprazole, provided initial confirmation of the pivotal functional role of PHOSPHO1 in skeletal mineralisation [30]. More recently, PHOSPHO1 deficient mice, *Phospho1*-R74X (*Phospho1* KO) were found to show elevated ePP*_i_* levels and to display multiple skeletal abnormalities, including spontaneous fractures, bowed long bones, osteomalacia and scoliosis in early life [31]. These pathological changes were clearly evident at 1 month of age in *Phospho1* KO mice, and this effect is thought to become progressively worse with age. Furthermore, tibiae from *Phospho1* KO mice are more ductile and did not fracture during 3-point bending but deformed plastically [32], [33], likely due to a reduced elastic modulus and hardness [32], [33].

Previous micro-computed tomography (μCT) analysis of 1-month-old *Phospho1* KO showed increased trabecular number and decreased trabecular space but no significant difference in BV/TV ratio compared to WT mice, along with a significant reduction in cortical mineral density in both femur and tibia [31]. Together, these findings suggest that PHOSPHO1 serves a critical role in bone mineralisation during development and growth. This, we have hypothesised, is related to its capacity to scavenge P*_i_* from both PEA and PCho in order to generate the P*_i_* concentration needed to establish a P*_i_*/PP*_i_* ratio permissive for the initial formation of HA crystal inside the MVs [3], [29].

To date, analyses of the *Phospho1* KO phenotype have been limited to young mice which are characterised by active modelling of the skeleton and as PHOSPHO1 has been implicated in the initiation of bone mineralisation, it is unclear whether this role is conserved in later life in the mature murine skeleton where bone turnover is limited [31], [32]. Furthermore, the level of mineralisation and the properties of the bone matrix are associated with bone strength and stiffness [34], [35], [36]. We therefore sought to determine how *Phospho1* contributes to tibial surface strain and stiffness using digital image correlation as reported previously [37]. Since matrix mineralisation has also been reported to influence terminal osteoblast differentiation into osteocytes [38], [39] and angiogenesis [40], [41], [42], [43], [44], [45], [46], it is also important to determine whether the osteoblast-to-osteocyte transition and vascular porosity [47], [48], [49], [50], [51] are impaired in PHOSPHO1 deficient mice as this may have profound effects on skeletal architecture and biomechanical properties due to an impaired ability of the skeleton to respond appropriately to mechanical loading [52], [53]. In this study, we have therefore used high resolution CT to examine the effect of *Phospho1* deletion on tibial bone architecture in mice at a range of ages to establish whether its role is conserved during growth and maturation of the skeleton. Furthermore, we have also explored whether the hypomineralised bones in these mice exhibit modified osteocytic and vascular content.

## 2 Materials and methods

### 2.1 Animal model

*Phospho1*-R74X-null mutant (*Phospho1* KO) mice were generated by N-ethyl-N-nitrosourea mutagenesis (ENU) as described previously [31]. Mice were housed up to 4 per cage in polypropylene cages with wood chip and paper bedding and provided standard mouse chow and water ad libitum throughout the study. Weaners up to 8 weeks of age were fed a standard rodent breeding diet and thereafter a standard rodent maintenance diet (Special Diet Services, South Witham, UK). All procedures complied with the UK Animals (Scientific Procedures) Act 1986 and were reviewed and approved by the ethics committee of The Roslin Institute, University of Edinburgh and the Royal Veterinary College (London, UK).

We chose to study the role of *Phospho1* in the maintenance of bone architecture at various life stages and therefore used male mice at 5 (young, early stage of growth), 7 (growing, later stage of growth), 16 (skeletally mature) and 34 (post-maturation) weeks of age.

### 2.2 Load-related tibial bone surface strains using digital image correlation

Digital image correlation (DIC) was used to describe strain distribution engendered by load application through points of articulation [37], [54]. Accordingly, male wild-type (WT) and *Phospho1* KO mice (n = 4/strain) at 5, 7 and 16 weeks old were euthanized, and right tibiae were exposed and covered with a thin layer of matt, water-based, white paint. Bones were subsequently speckled with matt, acrylic, black ink using a high precision air brush [54]. Legs were inserted in custom built loading cups attached to a material testing machine (Instron 5800, High Wycombe, UK) and loaded at a rate of 8 N/min up to 12 N/min. These cups ensured the bone to be loaded axially across the knee and ankle joints [55]. Two CCD cameras (100 mm lenses, GOM GmbH, Germany) mounted on a tripod at a reciprocal distance of 148 cm were positioned horizontally in front of the loading cups, at a distance of 42 cm, to provide a 15 × 12 mm field of view with 1.2 mm depth of focus. The two cameras were rotated towards each other meeting at 25° angle on the bone surface. A high-precision panel 15 mm × 12 mm was used to calibrate the system (GOM GmbH, Germany). The bone was illuminated by two diode lamps with polarised filters. During the loading, images of the medial side of the tibiae surface were recorded at 1 N interval using the ARAMIS 5M System (GOM GmbH, Germany), with a resolution of 7.5 × 10.9 μm. Post processing of the images was done using 19 × 19 pixel square facets, with 15 pixel step facet. Strains on the bone surface were computed with a computation size of 5 and a validity quote of 65%. Accuracy was determined at zero loading (zero strains) by taking three images in the un-deformed state during the experiments. Maximum and average strains on the medial surface were calculated at 12 N for all samples. The noise was consistent throughout all the tests and of approximately 0.03%.

### 2.3 High-resolution micro and nano-computed tomography

#### 2.3.1 μCT

Tibiae from 5-, 7- and 16- (n = 6/age group) as well as 34-week old (n = 5) *Phospho1* KO mice and WT were fixed in 70% EtOH and stored until scanning using the Skyscan 1172 (Skyscan, Kontich, Belgium), with X-ray tube operated at 50 kV and 200 μA, 1600 ms exposure time with a 0.5 mm aluminium filter and a voxel size of 5 μm. The scanning time for each sample was approximately 2 h. The slices were then reconstructed using NRecon 1.6.9.4 (Skyscan, Kontich, Belgium). 2D/3D analyses were performed using CTAn 1.13.5.1 + version software (Skyscan, Kontich, Belgium). CTVol 2.6.0 r98 version (Skyscan, Kontich, Belgium) was used for 3D visualisation. Phantom calibrated μCT was used to assess cortical tissue mineral density (TMD) on a stack of 100 slices for cortical region at 37% of total tibial length using two Skyscan-supplied bone phantoms with known mineral density values of 0.25 and 0.75 g/cm^3^ calcium hydroxyapatite. The phantoms were scanned and reconstructed with the same settings used to scan tibiae from WT and *Phospho1* KO mice.

#### 2.3.2 NanoCT

The tibial-fibula junction in 5-, 16-, and 34-week old (n = 4/age group) *Phospho1* KO mice and WT was scanned using the Skyscan 1172 (Skyscan, Kontich, Belgium) X-ray microtomograph. The samples were placed in Orthodontic Wax (Kerr, CA, USA) at 50 kV and 200 μA, 9800 ms exposure time with a 0.25 mm aluminium filter (99.999% purity, Goodfellow, Huntington, UK), voxel size of 0.6 μm, 360° at a rotation step of 0.25°. Two-frame averaging was used to improve the signal-to-noise ratio. The scan time for each sample was approximately 7 h. Prior to reconstruction, thermal shift in projection images was corrected in NRecon 1.6.9.4 (Skyscan, Kontich, Belgium). The slices were then reconstructed in NRecon using a ring correction factor of 15, smoothing of 1 and 35% beam hardening correction.

### 2.4 Morphometrical analysis

#### 2.4.1 Trabecular and positional cortical analysis

Prior to analysis, μ-CT images were re-oriented in DataViewer 1.5.0 (Skyscan, Kontich, Belgium), such that the cross-section within the transverse plane was perpendicular to the long axis of the bone. Tibial length was measured in CTAn 1.13.5.1 + software using a straight line measuring tool and the appearance of the trabecular ‘bridge’ connecting the two primary spongiosa bone ‘islands’ was set as reference point for analysis of the metaphyseal trabecular bone adjacent to the epiphyseal growth plate. 5% of the total bone length from this point (towards the diaphysis) was utilised for trabecular analysis of the proximal tibia. Cortical bone was analysed at one point along the bone shaft, at 37% of the total length (proximal-middle) from the reference starting slice (first appearance of medial tibial condyles). These areas were chosen in reference to previously published data on cancellous and cortical tibial bone [52], [56], [57]. As bones from different mice varied in length it was more useful to define a percentage of bone length for analysis of these regions in order to reduce undersampling/oversampling effects. The selected trabecular and cortical regions of interests were analysed using CTAn BatMan software (Skyscan, Kontich, Belgium) and morphometric parameters were recorded.

#### 2.4.2 Whole bone cortical analysis

Whole bone analysis was performed on datasets derived from whole CT scans using BoneJ [58] (version 1.13.14) a plugin for ImageJ [59]. Following segmentation, alignment and removal of fibula from the dataset, a minimum bone threshold was selected for each bone to separate higher density bone from soft tissues and air. The most proximal and the most distal 10% portions of tibial length were excluded from analysis, as these regions include the trabecular bone. This threshold was used in “Slice Geometry” within BoneJ plugin to calculate cross sectional area (CSA), second moment of area around minor axis (Imin), second moment of area around major axis (Imax) and mean thickness determined by local thickness in 2D (Mean Thick).

#### 2.4.3 NanoCT lacunae and canal analysis

300 consecutive images from the tibia–fibula junction were selected from each specimen. The images were loaded in CTAn software (Skyscan, Kontich, Belgium). Initially, foreground was segmented from background and a series of noise removal ‘despeckling’ steps were performed. Pores smaller than 13 μm^3^ and larger than 1500 μm^3^ were assumed to be noise and canals, respectively and the rest were considered to be lacunae. Osteocyte lacunar indices included average lacunar number (N.Lc), average volume (Lc.V), thickness (Lc.Th), separation (Lc.Sp), connectivity density (Lc.Con.Dnn) as well as canal indices including average canal number (Ca.N), average volume (Ca.V), thickness (Ca.Th), separation (Ca.Sp) and canal connectivity density (Ca.Con.Dnn) were calculated by measuring the 3D parameters of each discreet object within the volume of interest after segmentation. Shape analysis of the lacunae was conducted utilizing ‘Analyze Particles’ function in BoneJ. Shape parameters were then computed for each ellipsoid based upon the resulting three radii. The best-fit ellipsoid provided lacuna major radius (Lc.λ1), lacuna intermediate radius (Lc.λ2) and lacuna minor radius (Lc.λ3), which correspond to the lacuna's principal axes (i.e. the eigenvalues of the inertial matrix). These values allowed calculation of the degree of lacunar equancy (Lc.Eq >= Lc.λ3 / Lc.λ1), degree of lacunar elongation [Lc.El = 1 − (Lc.λ2 / Lc.λ1)] and degree of lacunar flatness [Lc.Fl >= 1 − (Lc.λ3 / Lc.λ2)] [60], [61]. The composition of the structure was then plotted using a Flinn diagram [62] showing major:intermediate axis ratio on the y-axis and the intermediate:minor axis ratio on the x-axis.

### 2.5 Primary osteoblast cultures

Primary calvarial osteoblast cells were isolated from 3 day-old WT and *Phospho1* KO mice [39]. Briefly, excised calvaria underwent sequential enzyme digestion [1 mg/ml collagenase type II (10 min); 1 mg/ml collagenase (30 min); 4 mM EDTA (10 min); 1 mg/ml collagenase (30 min)]. The cells were collected from each digest, re-suspended in α-MEM supplemented with 10% FBS and 50 μg/ml gentamicin and cultured at 37 °C with 5% CO_2_ until confluent. For experiments, cells were seeded at a density of 1.5 × 10^4^ cells/cm^2^. At confluency (day 0), growth medium was supplemented with 50 μg/ml ascorbic acid (Sigma) and 6 mM calcium chloride for up to 28 days to induce extracellular matrix mineralisation. The medium was changed every second/third day and samples collected at days 0, 7, 14, 21 and 28 of culture.

### 2.6 Assessment and quantification of mineralisation

Cell monolayers were fixed with 4% paraformaldehyde (PFA) for 5 min at 4 °C. After several washes in PBS, cells were stained with aqueous 2% (w/v) Alizarin red solution (Sigma) at pH 4.2, for 5 min at room temperature, before washing with water, to remove any unbound stain. The Alizarin red stain was subsequently solubilised in 10% cetylpyridinium chloride (Sigma) and the optical density of the resultant solution determined at 570 nm by spectrophotometry (Thermo Multiskan Ascent).

### 2.7 Real-time quantitative PCR (RT-qPCR)

RNA was extracted from primary osteoblast cell cultures using an RNeasy mini kit (Invitrogen) according to the manufacturer's instructions. For each sample, total RNA content was assessed by absorbance at 260 nm and purity by A260/A280 ratios, and then reverse-transcribed. RT-qPCR was performed using the SYBR green detection method on a Stratagene Mx3000P real-time qPCR system (Stratagene, CA, USA), or a LC480 instrument (Roche) as previously described. *Pdpn* primers were purchased from PrimerDesign Ltd, Southampton, UK (forward AAC AAG TCA CCC CAA TAG AGA TAA T, reverse CTA ACA AGA CGC CAA CTA TGA TTC). *Sost* primers were purchased from Qiagen (sequences not disclosed). Reactions were run in triplicate and routinely normalised against *Gapdh* (*PrimerDesign Ltd*. Sequences not disclosed).

### 2.8 Western blotting

The tibial diaphysis from 3 week old WT and *Phospho1* KO mice was snap-frozen in liquid nitrogen and stored at − 80 °C. Bones were subsequently ground in liquid nitrogen and then homogenised in 500 μl RIPA buffer (150 mM NaCl, 1.0% IGEPAL® CA-630, 0.5% sodium deoxycholate, 0.1% SDS, 50 mM Tris, pH 8.0) (Sigma) containing protease inhibitors (Roche). Lysates were frozen at − 20 °C. Protein concentrations were determined by a DC assay (Bio-Rad, Hemel Hempsted, UK) and 10 μg of protein was separated using a 10% bis-tris gel and then transferred to a nitrocellulose membrane and probed with goat anti-mouse E11 (1:1000, R&D Systems) and goat anti-mouse Sclerostin (1:500, R&D systems), followed by HRP-linked rabbit anti-goat secondary antibody (1:3000, Dako, Cambridge, UK), diluted in 5% non-fat milk (Marvel, Lincs UK). Membranes were washed in TBST and the immune complexes visualised by chemiluminescence using the ECL detection kit and an ECL film-based technique (GE Healthcare, Amersham, UK). Equal loading of protein was confirmed by stripping the blot in Restore Western stripping buffer (Pierce, Rockford, USA) for 30 min at 37 °C and subsequent re-probing with HRP-conjugated anti β-actin antibody (170000, Sigma). Densitometric analysis was performed using ImageJ Software (U. S. National Institutes of Health, Maryland, USA).

### 2.9 Statistical analysis

Statistical analyses were performed using either GraphPad Prism 6 (GraphPad Software, Inc., San Diego, CA) or “R”, version 3.1.1 (R Foundation for Statistical Computing, Vienna, Austria; http://www.r-project.org). Continuous measurements were summarised as means ± SEM. Linear model (two-way analysis of variance) was used to determine the effects of age (5, 7, 16 and 34 weeks) and genotype (WT and *Phospho1* KO) and their interaction on all phenotypic measurements and normality of residuals was assessed using the Shapiro–Wilk test. Bonferroni post-hoc correction was carried out for whole bone measurements, whilst no p-value adjustment was made on the post-hoc comparison for CSA, Imin, Imax and mean thickness from 10 to 90% tibial length. This was to preserve the original inferential statistics across the 10–90% tibial length and results were interpreted cautiously across tibial length. Statistical significance level was set at 5%.

## 3 Results

### 3.1 *Phospho1* deficiency reduces tibial average strain and stiffness in young, growing mice and this effect diminishes with skeletal maturation

**Fig. 1:**
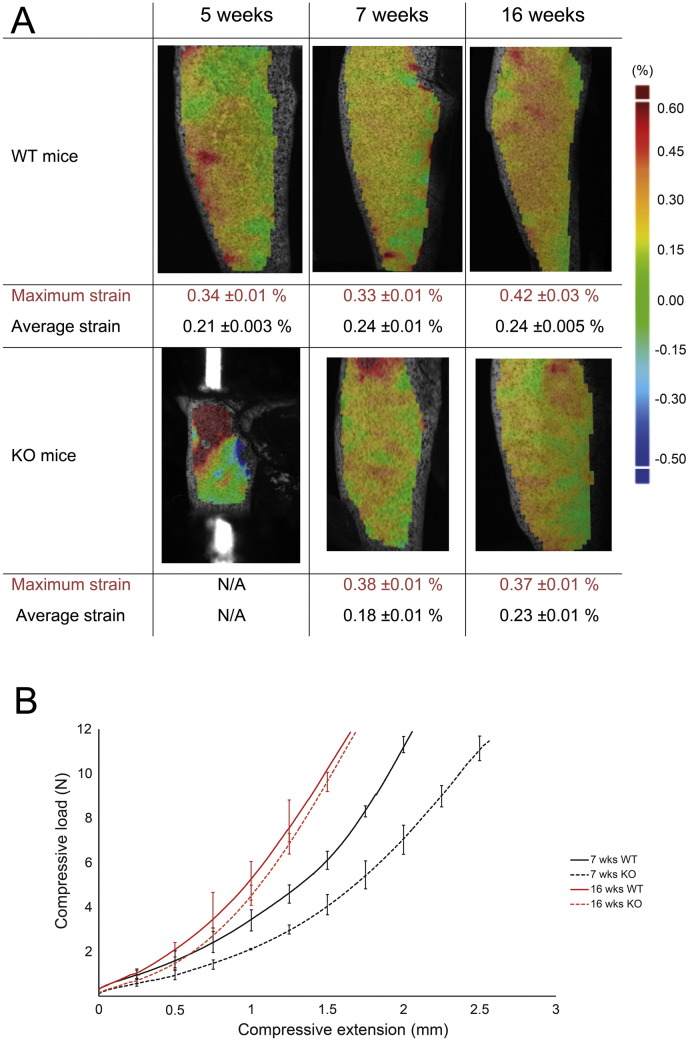
A) Longitudinal strain map on the medial side of the bone surface of WT and *Phospho1* KO tibia at 5, 7 and 16 weeks of age with maximum and average values obtained following 12 N compressive load. B) Loading displacement for 7 and 16 week old *Phospho1* KO and WT mice.

Spatial strain distribution was calculated across the medial tibia, based on the three components of displacement measured by the digital image correlation (DIC) system; however, Fig. 1 shows only strain in the axial (loading) direction, as transverse and shear strains both had relatively low magnitude in comparison. In agreement with previous studies [37], [54], axial compressive loads generated a non-uniform strain field across the surface of the tibia, with tension on the medial side because of its curved shape. It was not possible to detect strain distribution in tibiae from 5 week-old *Phospho1* KO mice due to their relatively small size (the cups were too close and the bone was not visible by the cameras, Fig. 1A).

Consistent with previous findings [32], [63], patterns of tissue strain showed that tibiae in *Phospho1* KO mice were more compliant at 5 and less so at 7 weeks of age than corresponding bones in WT group. However, average patterns of strain appeared to converge with advancing age, and were very similar in tibiae of WT and *Phospho1* KO at 16 weeks of age (Fig. 1A). Measurement of compressive displacement at varying load magnitudes supported these findings by showing that tibia of *Phospho1* KO mice were more compressible (25%) than WT mice at 7 weeks, under the same load (Fig. 1B). In contrast, compressive extension in *Phospho1* KO tibiae did not differ from WT at 16 weeks (Fig. 1B); both WT and *Phospho1* KO mice exhibited a maturation-related reduction in compressive displacement between 7 and 16 weeks of age and this modification was more marked in *Phospho1* KO mice (Fig. 1B). Thus, in line with previous studies [32], [63] we found that the bone of *Phospho1* KO mice is less stiff during growth and we also show that these differences in the degree of stiffness appear to be corrected upon attainment of skeletal maturity.

### 3.2 *Phospho1* deficiency generates age-related defects in trabecular bone, but produces compromised cortical bone architecture at all ages

**Fig. 2:**
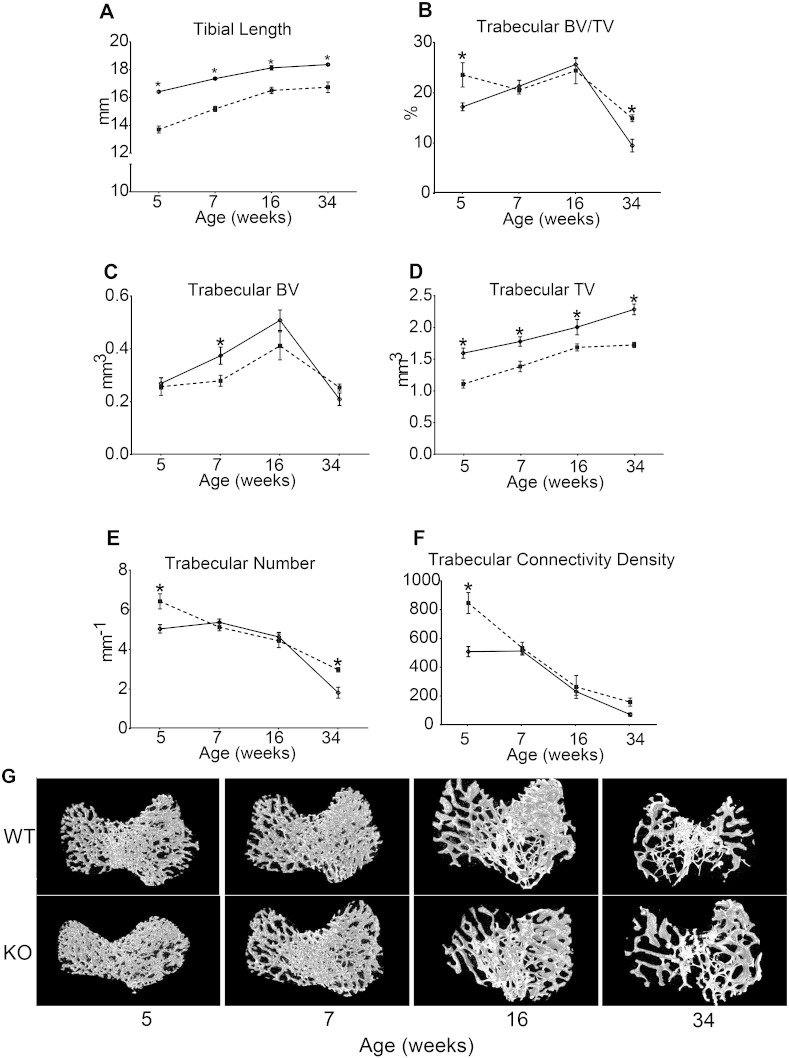
Trabecular bone phenotype of WT (solid) and *Phospho1* KO (dashed) tibia at 5, 7, 16 and 34 weeks of age. (*A*) Tibial length. Ex vivo high-resolution analyses of distal proximal metaphysical tibia to determine (*B*) trabecular bone volume/total volume (BV/TV), (*C*) trabecular bone volume (BV), (*D*) trabecular total volume (TV), (*E*) trabecular number, (*F*) trabecular connectivity density and (G) representative 3D μCT images of tibial trabecular bone in WT and KO mice. Linear graphs represent means ± SEM. Group sizes were *n* = 6 for 5-, 7- and 16- as well as n = 5 for 34-week old WT and KO mice. Statistical comparisons: p < 0.05 WT and KO of same age.

To explore whether these genotype-related differences in load-strain relationships are reflected in bone organisation, we have chosen specific ‘landmark’ locations along the tibia to analyse both trabecular and cortical bone architectures [52], [56], [57]. We find that *Phospho1* deficiency results in reduced tibial length in mice at all ages examined (Fig. 2A; p < 0.05), which was 17, 14, 10, and 11% shorter than WT tibia at 5, 7, 16 and 34 weeks respectively. These data are also consistent with greater age-related lengthening of tibiae in *Phospho1* KO compared to WT mice. Both age (p < 0.001) and genotype (p < 0.001) affect tibial length and an interaction between age and genotype is detected (Table 1; p < 0.05).

μCT based comparison of the tibial trabecular bone compartment revealed significantly higher BV/TV and trabecular number in *Phospho1* KO mice at 5 and 34 weeks of age (Fig. 2B; p < 0.05), but no significant differences in these parameters at either 7 or 16 weeks of age. This, however, would appear to mask the significantly lower bone volume in 7 week-old *Phospho1* KO mice (p < 0.05) which was not apparent at either 5 or 34 weeks of age and corresponds with lower total volume of the metaphyseal trabecular compartment in *Phospho1* KO than in WT mice at all ages (p < 0.05). There were no significant differences in trabecular thickness between WT and KO bones but, as expected, this increased with age (p < 0.001, Appendix 1). Trabecular connectivity density (Fig. 2F) was significantly greater in *Phospho1* KO mouse tibiae at 5 (p < 0.05) and somewhat elevated at 34 weeks, but no differences were apparent between KO and WT bones at 7 or 16 weeks of age.

Multiple comparisons across all groups revealed significant effects of *Phospho1* deficiency on trabecular total volume, number and connectivity density (p < 0.001; < 0.05 and < 0.01 respectively, Table 1) and significant interaction between genotype and age in trabecular number, connectivity density (p < 0.01; Table 1), trabecular separation and eccentricity (p < 0.05; Appendix 1). Together these analyses reveal that *Phospho1* deficiency leads to the elaboration of a smaller metaphyseal trabecular area at all ages, reduces BV at only some ages, and results in greater trabecular number at other ages, compared with WT mice. These data indicate that *Phospho1* deficiency leads to a defect in whole bone structure below the metaphyseal growth plate where age-related trends in trabecular architecture differ between the WT and *Phospho1* deficient bones.

**Fig. 3:**
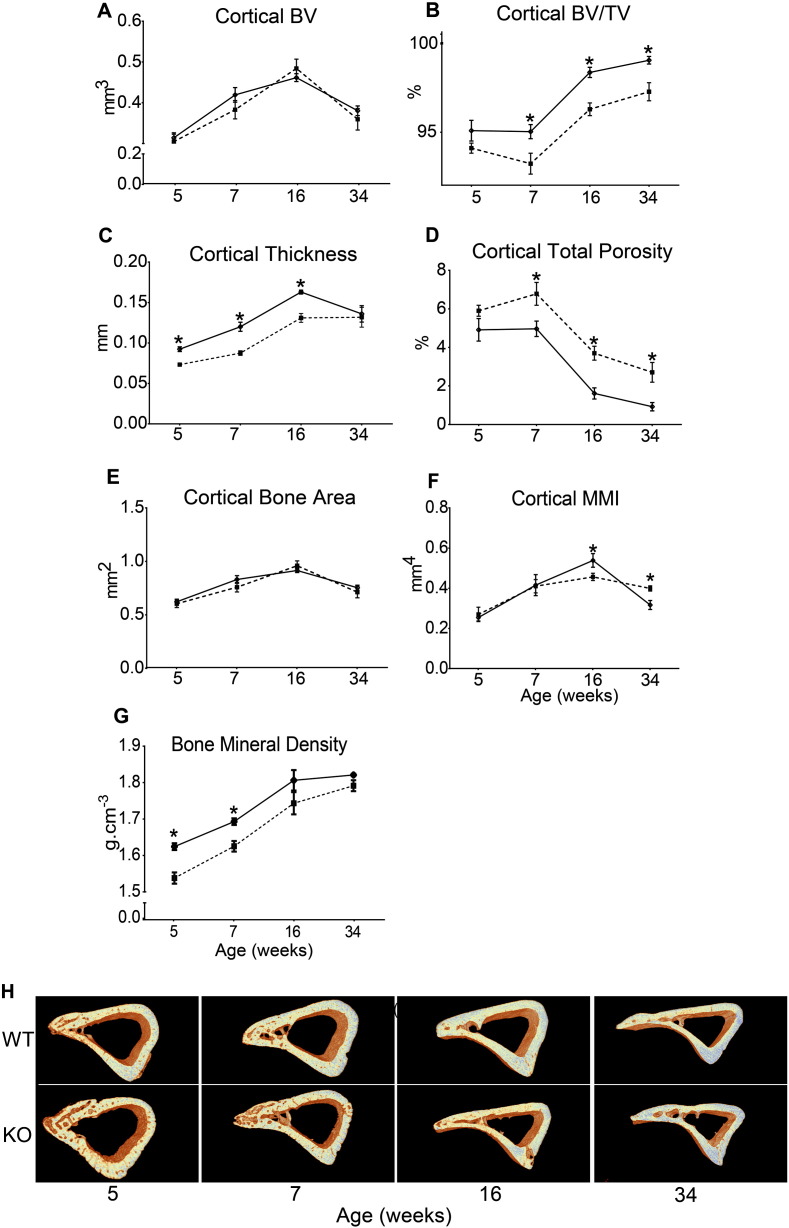
Cortical bone phenotype of WT (solid) and *Phospho1* KO (dashed) tibia at 5, 7, 16 and 34 weeks of age. *Ex vivo* high-resolution analyses of cortical bone at 37% of total tibial length showing (*A*) cortical total volume (TV), (*B*) cortical bone volume/total volume (BV/TV), (*C*) cortical cross sectional thickness, (*D*) cortical total porosity, (*E*) cortical degree of anisotropy, (*F*) cortical mean polar moment of inertia, (*G*) cortical tissue mineral density and (*H*) representative 3D μCT images of tibial cortical bone at 37% tibial length in WT and KO mice. Linear graphs represent means ± SEM. Group sizes were *n* = 6 for 5-, 7- and 16- as well as n = 5 for 34-week old WT and KO mice. Statistical comparisons: p < 0.05 WT and KO of same age.

A previous study reported reduced cortical mineral density and osteoid accumulation in the cortical bone of *Phospho1* KO mice compared with WT [32]. Our examination of the cortical bone at 37% of the total tibia length in *Phospho1* KO and WT mice shows that cortical bone volume was not modified by *Phospho1* deficiency at any of the ages studied; post-hoc analysis did, however, reveal expected age-related trends (p < 0.001; Fig. 3A). Age-related trends were also evident in TV (p < 0.001; Appendix 1) but this did not appear to be modified in *Phospho1* KO mice. In contrast, bone volume/total volume was significantly lower in bones of *Phospho1* KO mice older than 5 weeks (7, 16 and 34 weeks; Fig. 3B; p < 0.05). Thus, genotype (p < 0.001; Table 1) and age (p < 0.001; Table 1) both affected BV/TV but these did not exhibit interaction, supporting an age-independent effect of *Phospho1* deficiency.

Cortical cross sectional thickness was significantly lower (p < 0.01; Fig. 3C) in *Phospho1* KO mice at all ages, except at 34 weeks. Furthermore, both genotype and age affected cortical thickness (p < 0.001) but no interaction was found. Cortical total porosity was significantly higher in *Phospho1* KO bones at 7, 16 and 34 weeks (p < 0.001; Fig. 3D) and no interaction between genotype and age was detected. Moreover, our data show that *Phospho1* deficiency did not affect cortical bone area at any age (Fig. 3E). No differences in mean polar moment of inertia were observed in *Phospho1* KO mice at 5 or 7 weeks but levels were higher compared to WT at 16 weeks and lower at 34 weeks (p < 0.05; Fig. 3F). Thus, cortical analysis at this particular location along the tibial shaft suggested that *Phospho1* deficiency compromised cortical microarchitecture and leads to greater cortical porosity at all ages. Furthermore, our data show that TMD was significantly lower at 5 and 7 weeks in *Phospho1* deficient mice (p < 0.05; Fig. 3G) but no differences were observed at 16 and 34 weeks of age.

### 3.3 *Phospho1* deficiency produces age-related architectural changes in gross tibial anatomy and generates marked structural anomalies not observed by conventional analysis

To determine whether these cortical bone deficiencies at this particular location in *Phospho1* KO mice were generalised along the tibial shaft, we undertook whole-bone cortical analysis. We excluded the first and last 10% of total length, where there was significant trabecular bone volume, and removed the fibula by manual segmentation. Medians of the residuals normality test p-values along the % tibial length were 0.477, 0.157, 0.104 and 0.111 for CSA, Imin, Imax and Ct.Th, respectively.

**Fig. 4:**
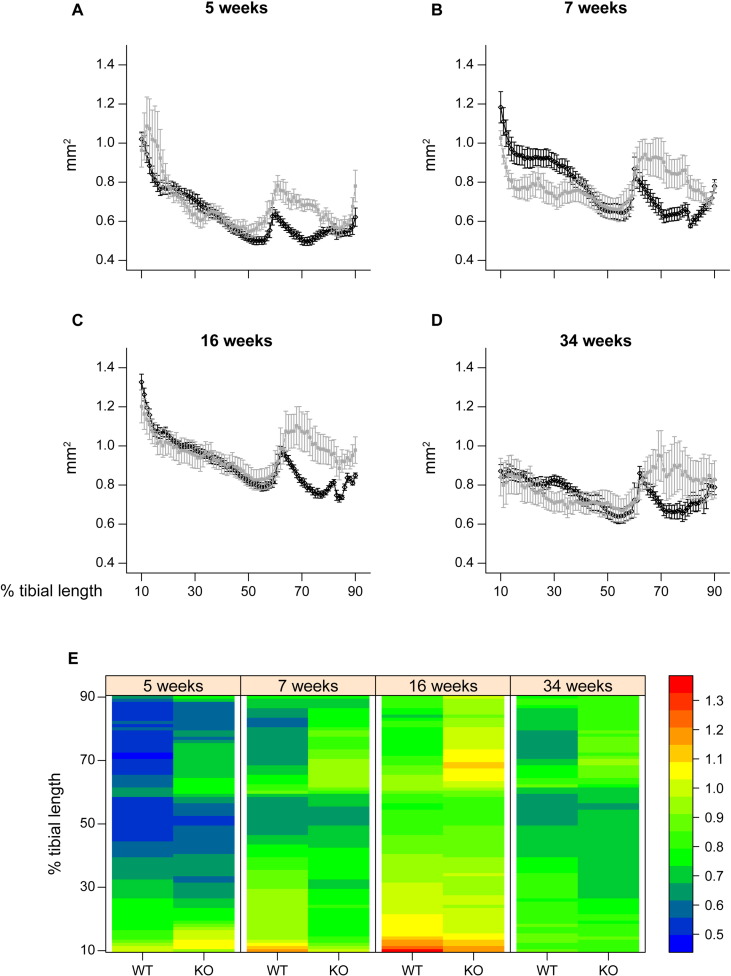
Cross sectional area (CSA) of WT (black) and *Phospho1* KO (grey) tibia at 5, 7, 16 and 34 weeks of age. Whole bone analyses of cortical bone between 10–90% of total tibial length, excluding proximal and distal metaphyseal bone showing cross sectional area at (*A*) 5 weeks, (*B*) 7 weeks, (*C*) 16 weeks and (*D*) 34 weeks. Line graphs represent means ± SEM. Group sizes were *n* = 6 for 5-, 7- and 16- as well as n = 5 for 34-week old WT and KO mice. (E) Graphical heat map representation of average tibial cross sectional area.

Our examination found that cross-sectional area (CSA) was significantly higher in KO compared to WT tibiae near the distal tibia at all ages (Fig. 4; 60–90%). In contrast, CSA was lower in proximal regions (Fig. 4; ~ 20–35%) in 7 and 34 week old *Phospho1* KO mice. Despite this, no significant interaction between age and genotype was detected.

**Fig. 5:**
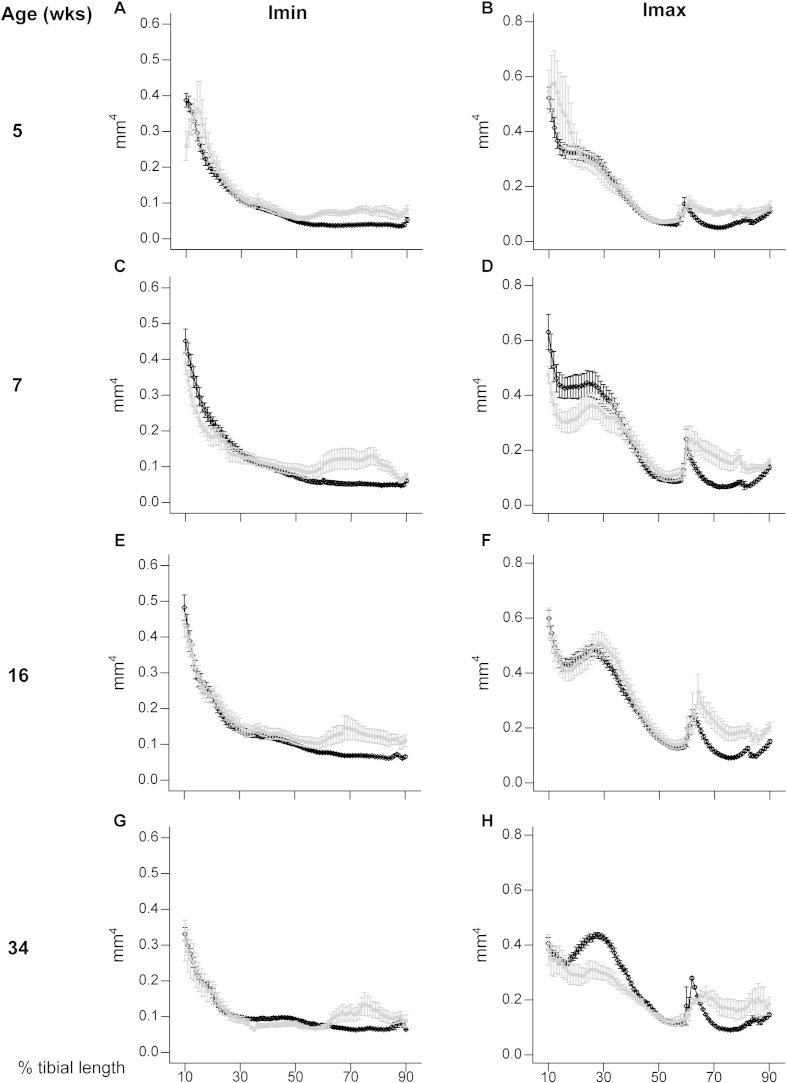
Minimum and maximum second moments of area (Imin and I max respectively) of WT (black) and *Phospho1* KO (grey) tibia at 5, 7, 16 and 34 weeks of age. Whole bone analyses of cortical bone between 10–90% of total tibial length, excluding proximal and distal metaphyseal bone showing Imin and Imax at (*A*, *B*) 5 weeks, (*C*, *D*) 7 weeks, (*E*, *F*) 16 weeks and (*G*, *H*) 34 weeks. Line graphs represent means ± SEM. Group sizes were *n* = 6 for 5-, 7- and 16- as well as n = 5 for 34-week old WT and KO mice.

To provide an estimate of tibial resistance to bending forces, we also calculated the second moment of area around minor (Imin) and major axes (Imax). These data show that Imin and Imax in *Phospho1* KO tibiae deviate from WT patterns and are significantly greater toward the distal tibia at all ages; no genotype: age interaction was detected (Fig. 5). In contrast, Imax is lower in proximal regions of the tibia of *Phospho1* KO mice. Together, CSA, Imin and Imax indicate that *Phospho1* deficiency produces tibial architecture likely reflecting regionalised changes in bending resistance at all ages, which is lower in proximal regions in 7 and 34 week-old mice and greater in the distal third at all ages (Fig. 7).

**Fig. 6:**
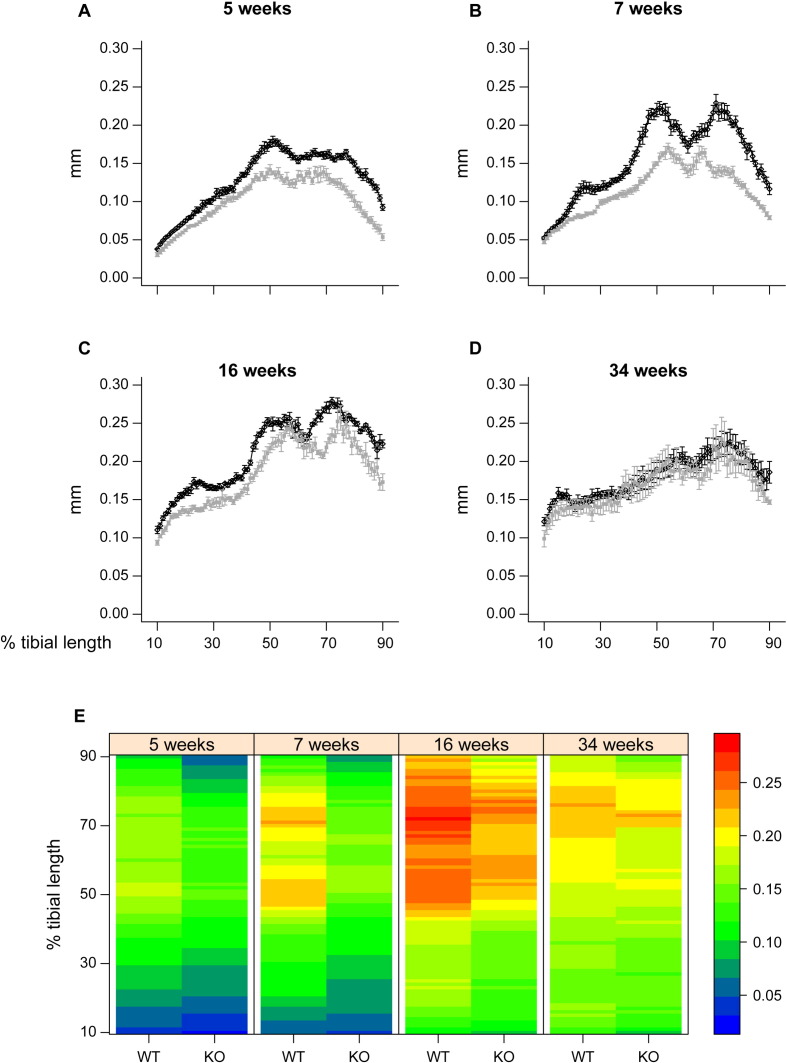
Mean cortical thickness of WT (black) and *Phospho1* KO (grey) tibia at 5, 7, 16 and 34 weeks of age. Whole bone analyses of cortical bone between 10–90% of total tibial length, excluding proximal and distal metaphyseal bone showing mean cortical thickness at (*A*) 5 weeks, (*B*) 7 weeks, (*C*) 16 weeks and (*D*) 34 weeks. Line graphs represent means ± SEM. Group sizes were *n* = 6 for 5-, 7- and 16- as well as n = 5 for 34-week old WT and KO mice. (E) Graphical heat map representation of average tibial mean cortical thickness.

**Fig. 7:**
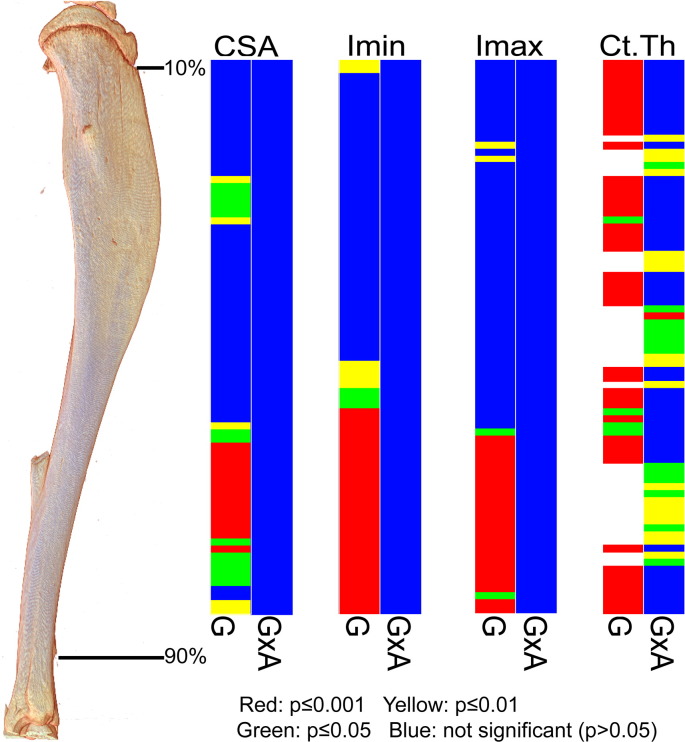
Graphical heat map representation of statistical significance of the effect of genotype (*Phospho1* deficiency) (G) and its interaction with age (GxA) on CSA, Imin, Imax and Ct.Th of tibia between 10 and 90% of length. Red p ≤ 0.000–0.001, yellow p ≤ 0.001–0.01, green p ≤ 0.01–0.05 and blue p > 0.05.

*Phospho1* KO mice also exhibited lower cross sectional thickness in almost all tibial regions at 5, 7 and 16 weeks, but did not differ from WT at 34 weeks of age (Fig. 6). Comparison across ages reveals that this lack of difference at 34 weeks is primarily due to greater decline in thickness with maturation/ageing in WT than in KO tibiae. These divergent age-related changes in cortical thickness in *Phospho1* KO tibiae contribute to significant age:genotype interactions (at locations along almost all the tibial length; Fig. 7). In addition to revealing greater utility of such whole bone analyses (that measurement at one location, 37%, may not necessarily always be representative), these data demonstrate that *Phospho1* deficiency produces proximodistally, regionalised modifications in indices of bending strength and cortical architecture, and leads to age-related changes in cross sectional thickness along the tibial length which diverge from WT.

### 3.4 *Phospho1* deficiency increases lacunar and vascular bone porosity

**Fig. 8:**
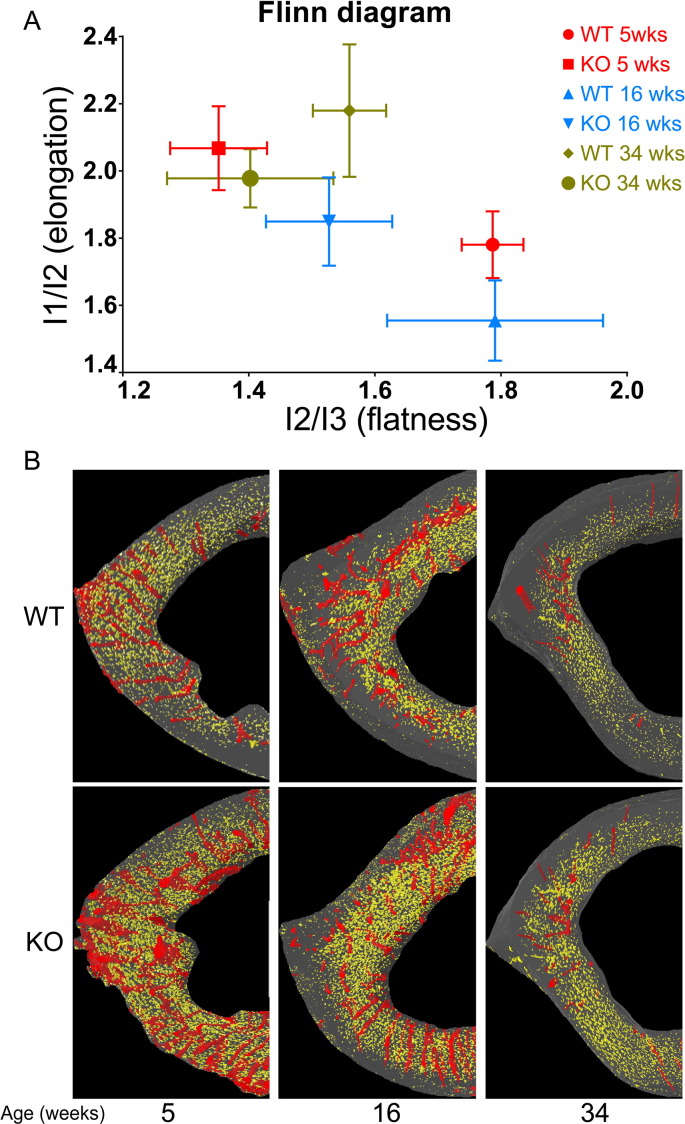
A) Flinn diagram displaying lacunar shapes in WT and *Phospho1* KO tibia at tibia–fibula junction from various ages. The x axis represents lacunar flatness which was calculated by dividing lacunar intermediate radius (l2: length of best-fit ellipsoid's intermediate radius) with lacunar minor radius (l3: length of best-fit ellipsoid's minor radius). The y axis represents lacunar elongation which was calculated by dividing lacunar major radius (l1: length of best-fit ellipsoid's major radius) with lacunar intermediate radius (l2: length of best-fit ellipsoid's intermediate radius). Data represent means with group sizes of *n* = 4 for WT and KO mice from different ages. B) Surface representation of the lacunar (yellow) and red (vascular porosity) segmented from 300 consecutive images from tibia–fibula junction from both genotypes and each age.

Regional control of bone architecture is thought to involve integration with the mechanical milieu by osteocytes and the process of mineralisation has been linked to osteocytogenesis [38], [39]. We therefore sought to determine whether *Phospho1* deficiency modifies osteocyte organisation, as well as vascular porosity, by measuring 3D morphometric parameters in high resolution images. Consistent with earlier analyses, we confirmed both lower TV and cortical thickness in *Phospho1* KO tibiae at all ages (Table 2). Our morphometric evaluation of the cortical bone at the tibia–fibula junction shows that porosity (or lacunar space) is greater in *Phospho1* KO than in WT bones, containing significantly greater numbers of osteocyte lacunae (N.Lc; p < 0.001; Table 2) with significantly greater volume (Lc.V; p < 0.001; Table 2). Whilst age also significantly impacts upon osteocyte lacunar number and volume, no interaction with genotype was observed. Intriguingly, analysis of lacunar shape showed that *Phospho1* KO bone contained osteocytes occupying somewhat elongated lacuna with significantly lower levels of lacunar flatness (Lc.El; p < 0.01). Based on these data, we have constructed a Flinn diagram [62] (Fig. 8) which shows that lacunar elongation and flatness were also both significantly affected post-maturation, but no age-related interaction with *Phospho1* deficiency was evident. Whilst *Phospho1* deficiency did not significantly alter lacunar equancy (Lc.Eq) in any age, relationships between lacunar elongation and flatness are plotted (Fig. 8) and support divergence in osteocyte shape in *Phospho1* deficient bone.

Greater porosity in *Phospho1* KO bone was also consistent with measures of vascular porosity, in which significantly higher canal number (N.Ca; p < 0.001 Table 2), density (N.Ca/Ct.TV; p < 0.05; Table 2) and volume (normalised by cortical tissue volume; Ca.V/Ct.TV; p < 0.05; Table 2) were evident in *Phospho1* KO bones. There was significant age-related decline in each of these parameters, and canal number showed significantly (p < 0.05) greater decline in *Phospho1* KO bone. These data indicate that the lower tissue volume and reduced cortical thickness at the tibia–fibula junction of *Phospho1* deficient mice are closely associated with increased porosity characterised by significant increases in the number of osteocytic lacuna and vessels.

### 3.5 *Phospho1* KO osteoblasts produce a hypomineralised matrix and show distinct osteocyte differentiation kinetics

**Fig. 9:**
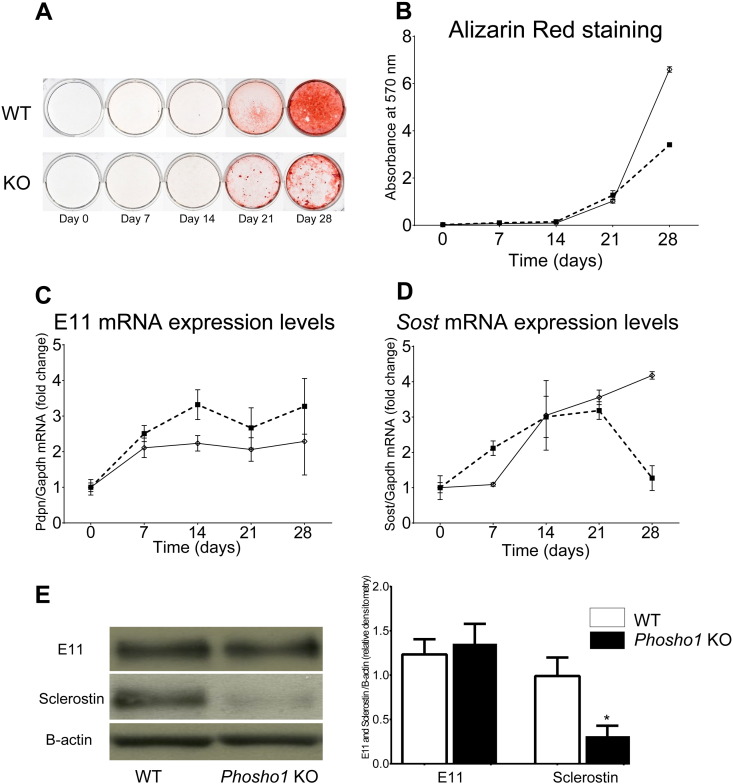
Characterisation of primary osteoblast-like cells isolated from WT and *Phospho1* KO mice to determine (A) mineralisation of cells, (B) *E11/Pdpn* mRNA expression levels, (D) *Sost* mRNA expression levels and (E) Western blots of E11 and sclerostin. For *in vitro* culture studies (A, B, C and D) results are the means ± SEM of three independent experiments (*n* = 4 per study). For Western blots (E) data represent means ± SEM with group sizes of *n* = 4 for WT and KO mice.

To explore whether these structural modifications and greater osteocyte lacunar numbers might reflect inherent characteristics of osteoblasts undergoing mineralisation in the absence of *Phospho1*, we examined the behaviour of primary osteoblasts from WT and *Phospho1* KO mice. Initial phenotypic characterisation of primary osteoblasts demonstrated significantly lower levels of matrix mineralisation in *Phospho1*-deficient primary osteoblast cultures than WT by days 21 and 28 of culture (p < 0.001; Fig. 9A/B). Despite reduced matrix mineralisation of *Phospho1* KO osteoblasts, RT-qPCR analysis showed that mRNA levels of E11/*Pdpn*, the early osteocyte marker, were significantly increased (vs WT) at time points beyond day 7 (p < 0.05; Fig. 9C), suggesting that osteoblast-to-osteocyte transition is nonetheless accelerated in the absence of PHOSPHO1. *Phospho1* KO osteoblast cultures also exhibited increased mRNA expression for *Sost*, a late osteocyte marker from day 7, and faster decline in these levels at day 28, compared to WT (p < 0.001; Fig. 9D). Western blot analysis of protein extracted from the tibia of 3 week old mice confirmed decreased sclerostin protein expression in *Phospho1* KO tibia in comparison to WT bones (p < 0.05; Fig. 9E). These data suggest that *Phospho1* exerts inherent effects on osteoblast characteristics and influences the osteocytogenic programme.

## 4 Discussion

Our data indicate that tibiae of *Phospho1* KO mice are less stiff during growth but rectified by skeletal maturation. Whole bone analyses show that *Phospho1* deficiency lowers proximal indices of bending resistance at only some ages, but that levels are consistently higher in distal regions at all ages. This is supported by data showing that *Phospho1* deficiency generates different age-related trends in whole bone structure below the growth plate in WT and *Phospho1* deficient bones, and compromised cortical microarchitecture with greater porosity at all ages. Our data reveal that modified *Phospho1* deficient bone morphology also encompasses marked alterations in osteocyte shape and significant increases in osteocytic lacuna and vessel number. The prospect that this is due to inherent deficits in osteoblast behaviour is bolstered by our data showing that primary *Phospho1* deficient osteoblast-like cells exhibit lower levels of matrix mineralisation and modified osteocytogenic programming *in vitro*.

Bone mineralisation is an essential and carefully controlled process for skeletal function and is regulated by both promoters and inhibitors of matrix mineralisation [64], [65]. Biomineralisation is dependent on PP*_i_*/P*_i_* homeostasis which is regulated by the actions of TNAP [10], [20], [21], [66], [67], NPP1 [11], [12], ANK [13], [14] and PHOSPHO1. Our knowledge of the involvement of these proteins in the regulation of biomineralisation and skeletal maintenance with maturation/ageing is incomplete. Furthermore, earlier studies did not extensively investigate the role of *Phospho1* in age-related maintenance of bone architecture and mechanical properties post-maturation.

A previous study reported that *Phospho1* deficiency resulted in increased trabecular number and decreased trabecular space in tibia [31]. This reported gain in trabecular bone mass was, in further studies, ascribed to a structural support role rectifying for the observed weaker (thinner and more porous) cortex [32]. However, this apparent compensation does not match with mechanical properties and greater incidence of greenstick fracture observed in *Phospho1* KO mice, nor does it provide an explanation for why the effects of *Phospho1*-deficiency differ in the trabecular and cortical compartments. Herein, we extensively investigate the role of *Phospho1* deletion on bone architecture with ageing.

In agreement with a previous study [32], [54] our DIC and load-deformation data showed that at younger age, *Phospho1* KO tibia was less stiff and was more compressible (25%) than WT bones; this effect was however diminished in 16-week-old skeletally mature mice, suggesting an age-dependent role on mechanical properties of *Phospho1*. As described previously [31], [32], [33], *Phospho1* deficiency results in a lower accumulation of mineral in bones, that consequently, lowers stiffness resulting in a more deformable bone. This more ductile bone is consistent with the protection of long bones from *Phospho1* KO mice against fracture during 3-point bending (36). This greater deformability in *Phospho1* KO mice is supported by the bowing of long bones from an early juvenile age and by fractures exhibiting predominantly green-stick presentation [31]. Although we have not tested bending properties of mice at 34 weeks of age, our data suggest that PHOSPHO1 serves a relatively limited role in the biomechanics of the mature skeleton and that biomechanical deficiencies due to the absence of PHOSPHO1 during growth and development are eventually corrected (in later life) by alternative mechanisms. Whether differences in cortical porosity and polar moment of inertia, evident at 34 weeks, will be retained when mice become even older remains to be elucidated in future studies. The mechanism(s) for these age-related corrections in average strain and stiffness are unclear but alternative mineralisation mechanisms clearly exist as the complete ablation of PHOSPHO1 function only leads to a decrease in the calcification ability of MVs but not to a complete lack of calcification [31], [32]. It is feasible that whilst the generation of P*i* within MVs through the actions of PHOSPHO1 is optimum for rapid and timely ECM mineralisation in early growth of the skeleton, this process can be mimicked, through time, by the influx of TNAP generated eP*_i_* into MVs via the phosphate transporter, P*_i_*T1 [3] in later life.

Our previous studies have also disclosed that the impaired ECM mineralisation noted in *Phospho1* KO mice may be in part explained by elevated osteopontin but not PP*_i_* levels. Indeed, the ablation of osteopontin improves the skeletal phenotype of *Phospho1* KO mice [68]. Whether differences in osteopontin expression between young and older PHOSPHO1 deficient mice can explain the correction in bone phenotype with age is unknown and requires further examination.

Our positional μCT analysis indicates, in agreement with a previous study [31], that *Phospho1* deficiency resulted in increased trabecular number, despite relative reductions in trabecular separation not reaching statistical significance. This observation without considering other structural parameters, such as BV and TV, suggests that *Phospho1* acts as a negative regulator of trabecular bone and hence in its absence, more trabeculae are formed. Alternatively, it is possible that greater bone formation occurs in order to compensate for the bone's relatively hypomineralised status. In this study, we report that despite smaller proximal metaphyseal volume in KO group in all ages, BV was similar to WT mice at 5 and 34 weeks of age and therefore increased BV/TV at these ages is a reflection of smaller trabecular total volume. Moreover, in agreement with Yadav et al. [9] cortical thickness was lower at 5 weeks and here we report that this effect is maintained through growth and adulthood but disappears in post-maturation group. In contrast, total cortical porosity was not different at 5 weeks but consistently higher in KO genotype in post-maturation groups. This type of positional analysis of both trabecular and cortical compartments suggests a transitory role of PHOSPHO1 in the maintenance of the trabecular bone and a more consistent role in cortical compartment.

The deficiencies observed using conventional analysis provided information about the effect of *Phospho1* deficiency in a short segment of the cortical bone. Non-biased whole-bone analysis revealed that the changes described at 37% of the tibial length were not necessarily representative of changes elsewhere in the bone. Several parameters of bone strength were measured. The estimated strength and rigidity of the bone can be determined through the study of the bone's cross sectional geometry which includes cortical cross sectional area (CSA) and second moments of area. CSA is directly related to a bone's strength against compressive forces applied equally throughout the bone; factors such as bone shape and the effects of muscle contraction however result in long bones experiencing bending and torsional forces. Herein indices of rigidity, including maximum and minimum resistance against bending forces in the cross section, second moment of area around minor axis (Imin) and second moment of area around major axis (Imax) [69], were also measured in WT and *Phospho1* KO bones at all ages. Our data from this approach reveal greater CSA, Imin and Imax throughout the distal third (~ 60–90%) of the tibia from *Phospho1* KO mice. In contrast, *Phospho1* deficiency lowered CSA in regions of the cortex closer to the proximal end of the tibia. These contrasting differences between the effect of *Phospho1* deficiency on the proximal and distal compartments of the tibia may indicate differential role for *Phospho1* during bone development, growth and skeletal maturity or that responses in these regions differ due to their relative undermineralisation.

Previous studies have described the relationship between osteocytes and their lacunae in humans and non-humans with mineralisation status; however, the quantification of 3D osteocyte density and morphology has always been problematic due to the relatively small number of osteocyte lacunae which cannot be visualised readily using traditional techniques including confocal microscopy. Large-scale analyses of osteocyte lacunar parameters in 3D will provide clarification on the relationship between mineralisation and osteocyte density *in vivo*. A number of previous studies [70], [71], [72] found no correlation between lacunar density and bone formation, architecture or resorption. In contrast, many other studies suggested that age [73], mechanical environment [51], [74], diet [75], [76] and glucocorticoid treatment [77] significantly affect lacunar morphology and density. It has also been reported that modification of loading environment leads to significant reduction in lacunar density [78]. Furthermore, Vashishth et al. [79] found positive correlations between both cortical and cancellous bone mass accrual and lacunar density. These inconsistent correlations between lacunar density and structural parameters indicate that variation in lacunar density may not be the major determinant of bone quality, although further in depth studies are needed to confirm these observations. Herein, we report that *Phospho1* deficiency significantly increases lacunar density and this effect remains after maturation. Furthermore, a similar observation was made with regard to vasculature content. These data suggest that osteoblast-to-osteocyte transition may be accelerated in the absence of PHOSPHO1. This acceleration is consistent with our *in vitro* observations in which osteoblasts from *Phospho1*-deficient mice exhibit elevated levels of E11/*Pdpn* mRNA (the early osteocyte marker) at initial stages, yet reduced levels of *Sost* mRNA (a mature osteocyte marker) at later stages of culture. The increase in E11/*Pdpn* mRNA levels *in vitro* was not observed at protein levels *ex-vivo*, presumably due to its transitory elevation, however, more stable reduction in *Sost* mRNA expression *in vitro* was confirmed at protein level in bones from *Phospho1* KO and WT mice. These data suggest that PHOSPHO1 negatively regulates osteocytogenesis and vascular porosity and in its absence both of these processes are upregulated.

Moreover, we analysed lacunar morphology to dissect the possible role of *Phospho1* in regulation of lacunar shape. Using confocal microscopy, McCreadie et al. reported that lacunar shape or size was not different between older women, with and without osteoporotic fracture [80]. In contrast, using high-resolution nanoCT, van Hove et al. suggested that osteocyte morphology in the subchondral cortical bone of the lateral articular surface of the proximal tibia obtained from osteoarthritic, osteopenic, and osteopetrotic patients was significantly different, which the authors attributed to their disease state [81]. Herein, we report that *Phospho1* deficiency alters lacunar shape with lacunae from KO mice exhibiting a more elongated shape compared to flat lacunae from the WT group. The shape of these osteocyte lacunae in WT mouse bone only drifted towards those shapes evident in the *Phospho1* KO mice once maturation had been reached.

Together our data suggest that deficiency in PHOSPHO1 exerts modifications in bone architecture that are transient and depend upon age, yet produces consistent modification in osteocyte differentiation and vascular porosity. It is possible that the inhibitory role of PHOSPHO1 on osteocyte differentiation leads to these age-related changes in bone architecture. It is also intriguing to note that this apparent acceleration in osteocyte differentiation evident in the hypomineralised bones of *Phospho1* KO mice suggests an uncoupling of the interplay between osteocytogenesis and biomineralisation. Further studies are required to dissect the molecular processes underlying the regulatory influences exerted by PHOSPHO1 on the skeleton with ageing.

## Figures and Tables

**Appendix 1:** Bone phenotype parameters of male WT and *Phospho1* KO mice at 5, 7, 16 and 34 weeks of age detailing post-hoc comparisons for effects of age and genotype and significant age:genotype interactions. Data shown are the means ± SEM.

	WT 5 weeks n = 7	KO 5 weeks n = 7	WT 7 weeks n = 7	KO 7 weeks n = 7	WT 16 weeks n = 7	KO 16 weeks n = 8	KO 37 weeks n = 5	KO 37 weeks n = 5	Effect of genotype	Effect of age	Interaction Age ∗ genotype
*Trabecular bone*											
Tb.Th (mm)	0.033 ± 0.000	0.035 ± 0.002	0.038 ± 0.001	0.039 ± 0.000	0.054 ± 0.001	0.053 ± 0.002	0.052 ± 0.001	0.049 ± 0.000	NS	< 0.001	NS
Tb.Sp (mm)	0.134 ± 0.005	0.120 ± 0.004	0.122 ± 0.003	0.131 ± 0.003	0.143 ± 0.005	0.148 ± 0.008	0.279 ± 0.042	0.206 ± 0.008	NS	< 0.001	< 0.05
Tb.DA (mm^− 1^)	2.640 ± 0.079	2.454 ± 0.143	2.614 ± 0.090	2.418 ± 0.095	2.306 ± 0.043	2.290 ± 0.098	1.863 ± 0.094	1.823 ± 0.066	NS	< 0.001	NS
Tb.Ecc (mm)	0.667 ± 0.039	0.760 ± 0.029	0.756 ± 0.012	0.686 ± 0.020	0.680 ± 0.021	0.658 ± 0.024	0.767 ± 0.036	0.684 ± 0.041	NS	NS	< 0.05

*Cortical bone*											
Tissue volume (TV)	0.328 ± 0.013	0.328 ± 0.321	0.439 ± 0.018	0.409 ± 0.025	0.468 ± 0.010	0.500 ± 0.022	0.382 ± 0.012	0.368 ± 0.026	NS	NS	< 0.001
Bone surface/bone volume (mm^− 1^)	31.153 ± 0.991	40.737 ± 1.111	24.963 ± 1.169	33.753 ± 1.043	18.684 ± 0.234	23.684 ± 0.620	21.318 ± 1.551	22.573 ± 1.997	< 0.001	< 0.001	< 0.01
Cortical eccentricity	0.698 ± 0.010	0.676 ± 0.015	0.487 ± 0.010	0.771 ± 0.015	0.796 ± 0.017	0.786 ± 0.016	0.843 ± 0.004	0.833 ± 0.005	NS	< 0.001	NS

**Table 1:** Results from the ANOVA, testing the significance of the main effects of genotype and age and their interactions between WT and *Phospho1* KO in metaphyseal trabecular and cortical bone.

Parameters	Genotype	Age	Genotype ∗ age
Tibial length	< 0.001	< 0.001	< 0.05
Trabecular bone			
BV/TV (%)	NS	< 0.001	NS
BV (mm^3^)	NS	< 0.001	NS
TV	< 0.001	< 0.001	NS
Tb.N	< 0.05	< 0.001	< 0.01
Conn.Dn (mm)	< 0.01	< 0.001	< 0.01
Cortical bone			
BV/TV (%)	< 0.001	< 0.001	NS
BV (mm^3^)	NS	NS	< 0.001
Ct.Th	< 0.001	< 0.001	NS
Tot.Po %	< 0.001	< 0.001	NS
B.Ar	NS	NS	< 0.001
MMI	NS	< 0.001	NS
TMD	< 0.001	< 0.001	NS

**Table 2:** Porosity parameters representing lacuna and vascular porosity of male WT and *Phospho1* KO mice at 5, 7, 16 and 34 weeks of age, detailing post-hoc comparisons for significant age:genotype interactions. Data represent means ± SEM with group sizes of *n* = 4 for WT and *Phospho1* KO mice from different ages.

Morphometric index	WT 5 weeks n = 4	KO 5 weeks n = 4	WT 16 weeks n = 4	KO 16 weeks n = 4	WT 34 weeks n = 4	KO 34 weeks n = 4	Effect of genotype	Effect of age	Interaction age ∗ genotype
*Bone parameters*									
Ct.TV (mm^− 3^)	0.085 ± 0.005	0.157 ± 0.002	0.140 ± 0.005	0.180 ± 0.012	0.117 ± 0.009	0.132 ± 0.022	< 0.01	< 0.01	NS
Ct.Th (mm)	0.089 ± 0.000	0.063 ± 0.002	0.119 ± 0.007	0.114 ± 0.011	0.144 ± 0.007	0.124 ± 0.003	< 0.01	< 0.001	NS

*Canal parameters*									
N.Ca	74 ± 8.495	282 ± 54.580	62 ± 13.444	136 ± 44.434	20 ± 4.366	63 ± 22.595	< 0.001	< 0.01	< 0.05
N.Ca/Ct.TV (mm^− 3^)	895.7 ± 137.35	1928.8 ± 458.01	509 ± 93.76	715 ± 185.91	164 ± 34.26	430.6 ± 92.88	< 0.05	< 0.001	NS
Ca.V/Ct.TV (%)	1.266 ± 0.233	2.375 ± 0.558	1.033 ± 0.302	1.128 ± 0.228	0.142 ± 0.045	0.546 ± 0.141	< 0.05	< 0.001	NS

*Lacunae parameters*									
N.Lc	1707 ± 234.6	5900 ± 768.9	2930 ± 574.3	5367 ± 740	789 ± 373	2197 ± 573	< 0.001	< 0.001	NS
N.Lc/Ct.TV (mm^− 3^)	20,483 ± 3327	38,761 ± 5951	25,026 ± 1519	29,336 ± 2282	6276 ± 2830	15,917 ± 1629	< 0.01	< 0.001	NS
Lc.V/Ct.TV (%)	0.571 ± 0.103	0.995 ± 0.137	0.665 ± 0.033	0.675 ± 0.142	0.157 ± 0.060	0.404 ± 0.060	< 0.05	< 0.001	NS
< Lc.Eq >	0.316 ± 0.011	0.363 ± 0.023	0.367 ± 0.014	0.548 ± 0.196	0.394 ± 0.074	0.368 ± 0.023	NS	NS	NS
< Lc.El >	0.433 ± 0.031	0.511 ± 0.026	0.346 ± 0.050	0.450 ± 0.043	0.527 ± 0.049	0.491 ± 0.023	NS	< 0.05	NS
< Lc.Fl >	0.439 ± 0.014	0.253 ± 0.041	0.426 ± 0.060	0.337 ± 0.038	0.356 ± 0.024	0.267 ± 0.070	< 0.01	NS	NS
